# CFD simulation of gas pressure drop in porous packing for rotating packed beds (RPB) CO_2_ absorbers

**DOI:** 10.1007/s11356-022-20859-x

**Published:** 2022-05-23

**Authors:** Justyna Wojtasik-Malinowska, Maciej Jaskulski, Marcin Jaskulski

**Affiliations:** 1 Department of Environmental Engineering, Faculty of Process and Environmental Engineering, Lodz University of Technology, Wolczanska st. 213, 90-924 Lodz, Poland; 2 Faculty of Geographical Sciences, Institute of Urban Geography, Tourism Studies and Geoinformation, University of Lodz, Kopcinskiego st. 31, 90-142 Lodz, Poland

**Keywords:** CFD, RPB, Porous packing, Pressure drop, RANS, Turbulence models

## Abstract

Rotating packed bed (RPB) is a promising technology which can be used to intensify mass transfer in absorption processes. A better understanding of fluid dynamics is crucial to fill the gap in fundamental knowledge. Raising awareness on new technology and creating rules for process design and control are also very important. The experimental investigation of fluid in rotating beds is a very complex and difficult issue. What is more, the knowledge of the phase behavior in an RPB device is still insufficient. Therefore, an CFD (computational fluid dynamics) simulation is proposed as a tool for the study of gas phase flow inside porous packing. This study presents a three-dimensional numerical model for two fluid models: k-ε and RNG k-ε, for predicting dry pressure drop. The obtained simulation outcome was compared with the experimental results. The experimental dry pressure drop for porous packing was investigated for rotational speed in the range from 150 rpm to 1500 rpm and compared to the results from the CFD model. The comparison between the experimental and simulation results indicates very good consistency for the entire range of the rotational speed of interest. CFD modelling is recognised as an adequate tool leading to the better understanding of gas phase behaviour inside an RPB, filling an essential gap in our knowledge of the hydrodynamics of rotating packing, which allows to improve the design and performance of the process in RPB in terms of minimizing energy and material consumption.

## Introduction

In recent years, the industry has recognised the need to increase process effectiveness and decrease process costs. Available technologies are slowly approaching their limits and sometimes they cannot be improved to satisfy those requirements. Furthermore, the cost of improving conventional equipment—especially in separation technologies—is steadily increasing. In order to address all these needs, new types of equipment are constantly being developed. Several different technologies based on high centrifugal forces were proposed for different applications in the industry with surprisingly good results. This class of equipment is called HiGee Technology (from High Gravity) and involves a wide range of techniques based on centrifugal force, whereas traditional solutions are based on gravity force (Rao et al. 2004; Neumann et al. 2018).

Such a solution is the RPB (rotating packed bed) technology, in which the mass exchange process takes place under high gravity conditions (Ramshaw and Mallinson 1981). This equipment allows for the intensification of multiphase contacting, large throughput, and short residence (Kelleher and Fair 1996; Agarwal et al. 2010). Furthermore, better turbulent conditions lead to a higher mass transfer coefficient which allows the minimisation of the size of RBP units in comparison to traditional columns (Rao et al. 2004). Thanks to its unique design, RBP was determined to have significant advantages over traditional packed columns, and it can also be used for some processes that are nearly impossible to conduct in conventional reactors. This technology has been known for 30 years, but there are still some aspects of it that require further investigation. One of the most important is the understanding of fluid dynamics inside the rotor. This is much more complicated than in standard packed columns because of the annular shape of the rotor and because the flow regime changes with radial distance from the centre. Even if some mathematical models of the RPB unit hydrodynamics are already created, the majority is dedicated to specific unit sizes and shapes.

Nowadays, a lot of research has been carried out on RPBs in the following fields: (i) flow hydrodynamics (Zhang et al. 2020; Gładyszewski et al. 2021); (ii) mass transfer (Wang et al. 2019); (iii) liquid holdup (Zhou et al. 2010; Xie et al. 2017); (iv) micromixing (Wenzel and Górak 2018; Ouyang et al. 2019), (v) distillation (Agarwal et al. 2010; Mondal et al. 2012; Qammar et al. 2019); and (v) absorption (Wojtasik et al. 2019; Liu et al. 2021). Currently, RPBs have already been widely applied in many chemical processes, e.g. distillation, absorption, deaeration of liquids, production of hydrochlorous acid, solvent recovery, methylene diphenyl diisocyanate production, isobutylene isoprene rubber production, biodiesel production, removal of volatile organic compounds (VOCs) from waste gas streams and selective H_2_S removal (Neumann et al. 2018). The RPB is also a promising technology for CO_2_ capture in the post combustion of a power plant because of the possible reduction of installation size and energy demand and its highly efficient operation (Cheng et al. 2010; Chamchan et al. 2017).

In order to improve the design and optimization stage for processes conducted in RPB, it is necessary to better understand the behaviour of the phases within this equipment. Researchers need to learn more about single-phase and two-phase flows. Due to the complex structure of the RPB and the rotating packing, the experimental measurement of that behaviour is difficult; therefore the solution should be searched in numerical methods.

CFD (computational fluid dynamics) simulations of RPBs help in understanding the physical behaviour of gas and liquid and gas-liquid interactions and mechanisms and assist in the scale-up of the reactor from lab-scale to industrial scale. However, in the RPB field, there are only a few reports on the use of CFD simulation. It can be caused by the complexity of the RPB structure and packing, which additionally operate in high rotational frequencies. Llerena-Chavez and Larachi (Llerena-Chavez and Larachi 2009) develop the three-dimension model to investigate the impact of different locations of the gas inlet on the flow distribution inside the RPB. Yang et al. (Yang et al. 2015) proposed a three-dimensional single-phase flow CFD model where simulation results were validated with experimental data. Liu et al. (Liu et al. 2017) propose a three-dimension model to analyse gas phase behaviour in RPB with structured stainless steel wire mesh packing. Guo el al. (Guo et al. 2017) developed a three-dimension model to liquid phase behaviour inside the RPB. CFD modelling was also applied to the simulation of two-dimensional micromixing inside the RPB using Reynolds stress turbulence models (RSM) (Shi et al. 2013; Guo et al. 2016). In the last years, two-phase CFD models are presented (Yang et al. 2016; Lu et al. 2018; Ouyang et al. 2018). In recent years, it can be observed the interest in CFD modelling for RPB devices has increased due to the possibilities offered by this tool towards understanding the behaviour of the phases inside the RPB unit. The knowledge of the phase behavior in an RPB device is still insufficient; therefore it is necessary to investigate this field. CFD modelling approach allows to fill the gap in our knowledge about fluid hydrodynamics inside RPBs.

The aim of this work is prepared CFD simulation of the gas phase flow inside porous packing and then compare simulation results with experimental results for investigated range of rotor speeds and volumetric gas flow rates. To describe one phase flow in RPB, the three-dimensional numerical model for two fluid models, k-ε and RNG k-ε for predicting dry pressure drop, is presented. The obtained results are presented and analysed to describe one phase flow in RPB.

## CFD model

### Methodology

**Fig. 1:**
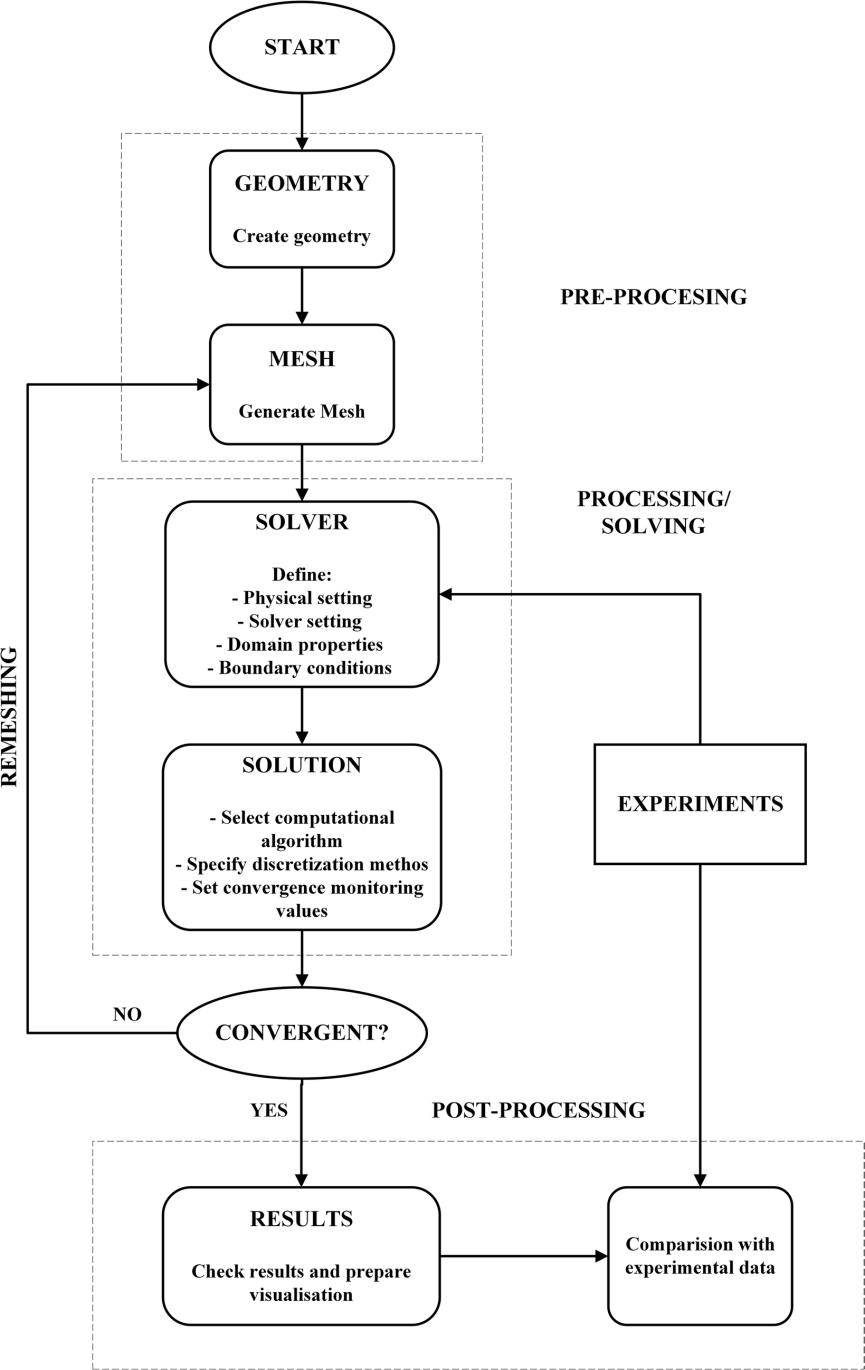
Flow chart of CFD simulation methodology

The numerical study is performed using the steps outlined in the flowchart in Fig. 1 for each simulation point. First, the geometry of the RPB unit is prepared. Then, a computational mesh was generated. For the prepared mesh, a domain was defined, and the properties of boundary conditions, domains and materials were determined. Some properties are described based on experimental results. The next stage of processing is the solution in which the CFD simulation results are calculated. If the calculations did not converge, it is returned to mesh preparation, if it does converge, the results are checked, and the visualization is prepared. In the last step, the obtained simulation results were compared with the experimental results and presented on common graphs. The individual stages of model preparation are described in more detail later in this article.

### Geometry

**Fig. 2:**
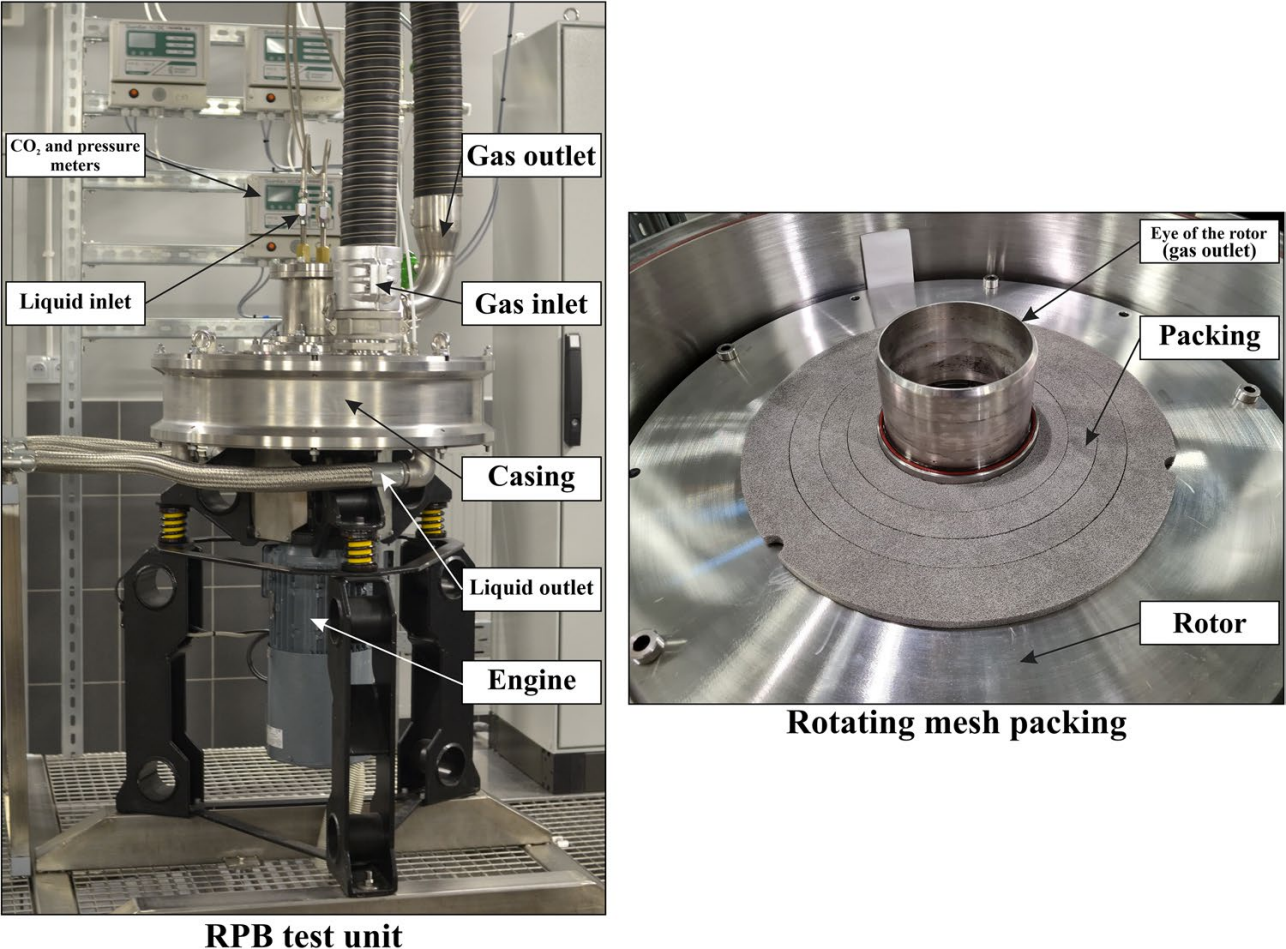
RPB unit and packing using as prototype to CFD simulations and generation of validation data

**Fig. 3:**
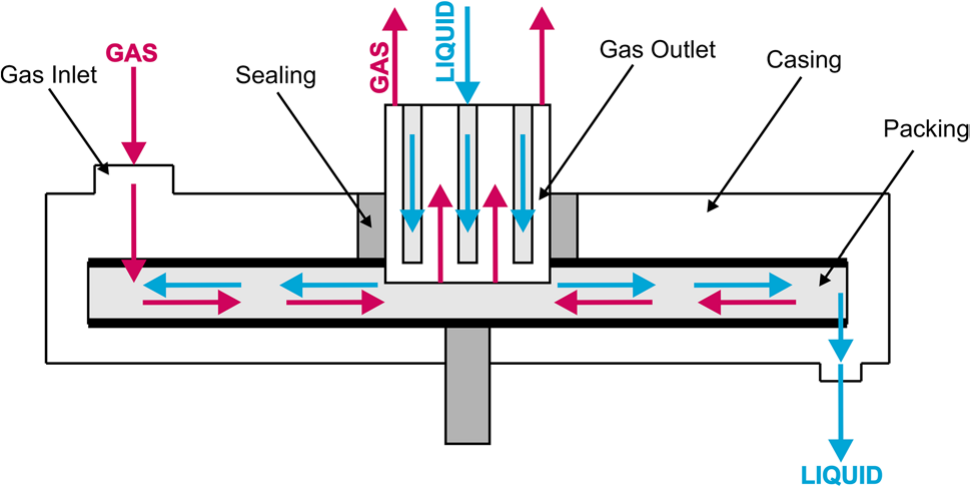
Phase contact diagram in RPB unit

The three-dimensional geometric model, used in the CFD simulation, reflects the real RPB unit constructed at the Lodz University of Technology. The RPB unit was designed to conduct research on the process of CO_2_ absorption in aqueous amine solutions. The asymmetric structure of the absorber makes it impossible to simplify the geometry to two dimensions or to a periodic object. The geometry does not include liquid outlets due to the preparation of a single-phase model for the gas phase. Air discharge through the liquid flow channels was not possible due to the use of a hydraulic closure. This allowed the geometry to be simplified without the risk of misrepresenting the real gas flow Fig. 2 and 3.

In this study, porous packing was used. The main dimensions of packing were the following: 10 mm height, 146 mm inner diameter, 600 mm outer diameter; the specific area was 2800 m^2^ m^-3^ and the porosity 0.922. The rotational speed of the bed varied from 150 rpm to 1500 rpm, and the gas flow rate ranged from 20 m^3^ h^-1^ to 60 m^3^ h^-1^. The described nickel-chromium foam packing was investigated experimentally in RPB, and the experimental and simulation results were compared.

### Computational mesh

The prepared geometry of the absorber was subjected to a discretisation process in order to generate a computational mesh. Due to disorderly air flow, it was decided to use a tetrahedral mesh. As gas flows through the porous layers, however, the wall effect on the flow pattern is significant. Thus, a boundary layer was generated in the rotating domain representing the porous body. It was necessary, however, to calculate the thickness of the first boundary layer element (*y*_*H*_) and the number of layers (*N*) that could describe the velocity gradient at the rotor walls (White 2009).

In the first step, the Reynolds number (*Re*) is calculated according to Eq. 1:

1 $$\mathit{\operatorname{Re}}=\frac{\rho vl}{\mu }$$

The characteristic geometric dimension *l* is represented by the width of the spacing of the rotor plates, *ρ* is the gas density, *v* is the gas velocity and *μ is the* gas dynamic viscosity. For the calculated Reynolds number, it is possible to determine the skin friction coefficient (*c*_*f*_) according to Eq. 2:

2 $${c}_f={\left(2\;{\mathit{\log}}_{10}\left(\mathit{\operatorname{Re}}\right)-0.65\right)}^{-2.3}$$

Having computed the skin friction coefficient, the wall shear stress (*τ*_*w*_) is calculated following Eq. (3):

3 $${\tau}_w=\frac{1}{2}\rho {v}^2{c}_f$$

The friction velocity (*u*_*τ*_) can then be calculated from the wall shear stress (Eq. 4):

4 $${u}_{\tau }=\sqrt{\frac{\tau_w}{\rho }}$$

Finally, the distance of the first calculation point from the rotor wall (*y*_*p*_) can be determined:

5 $${y}_p=\frac{y^{+}\mu }{u_{\tau}\rho }$$

As the boundary layer elements are prisms, the calculation point distance is half the thickness of the element (*y*_*p*_):

6 $${y}_H=2{y}_p$$

For the assumed value *y*^*+*^ *= 1*, the thickness of the first layer is equal to 7.22∙10^-4^ m.

In order to determine the number of elements of the boundary layer, it is necessary to calculate the thickness of the laminar sublayer *(δ)* using the Blasius corelation (White 2009):

7 $$\delta =4.64\frac{l}{\sqrt{\mathit{\operatorname{Re}}}}=4.64\sqrt{\frac{\vartheta l}{v}}$$

where *υ* is the kinematic viscosity of gas. If each element of the boundary layer is 20% (*G = 1.2*) wider than the previous one, the total thickness of the laminar sublayer can be expressed as a geometric sequence:

8 $$\delta ={y}_H+{y}_HG+{y}_H{G}^2+\dots {y}_H{G}^{N-1}$$

where *N* is the number of boundary mesh layers. The above geometric sequence can be written in abbreviated form as follows:

9 $$\delta ={y}_H\frac{1-{G}^N}{1-G}$$

By transforming the equation with respect to *N*, we obtained Eq. (10) for the number of layers:

10 $$N=\frac{\mathit{\log}\left(\frac{\delta \left(G-1\right)}{y_H}+1\right)}{\mathit{\log}(G)}$$

Our calculations show that for the highest Reynolds numbers that can occur during gas flow, we need a boundary layer composed of seven sublayers.

**Fig. 4:**
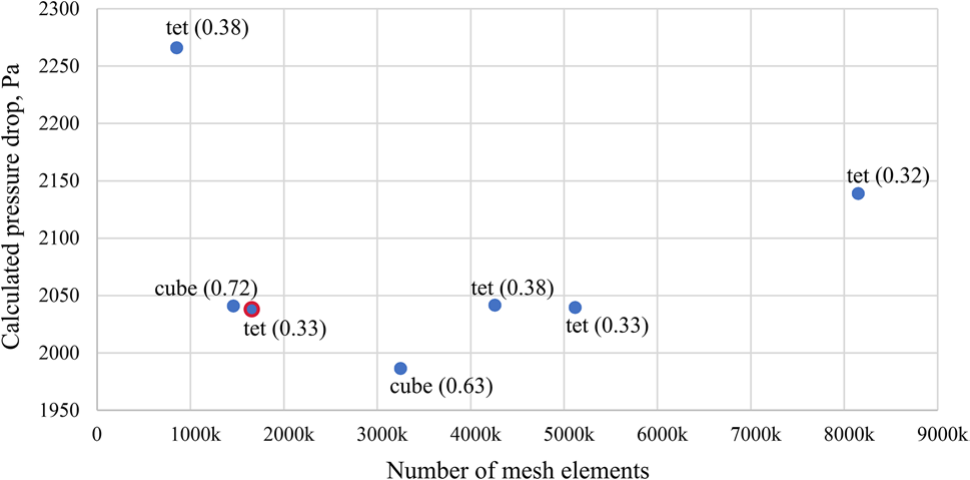
The result of the test of the independence of calculations from the mesh density. Cube, cubic mesh; Tet, tetrahedral mesh. The values in parentheses indicate the average skewness of the elements. The grid used in the calculations is marked in red

**Fig. 5:**
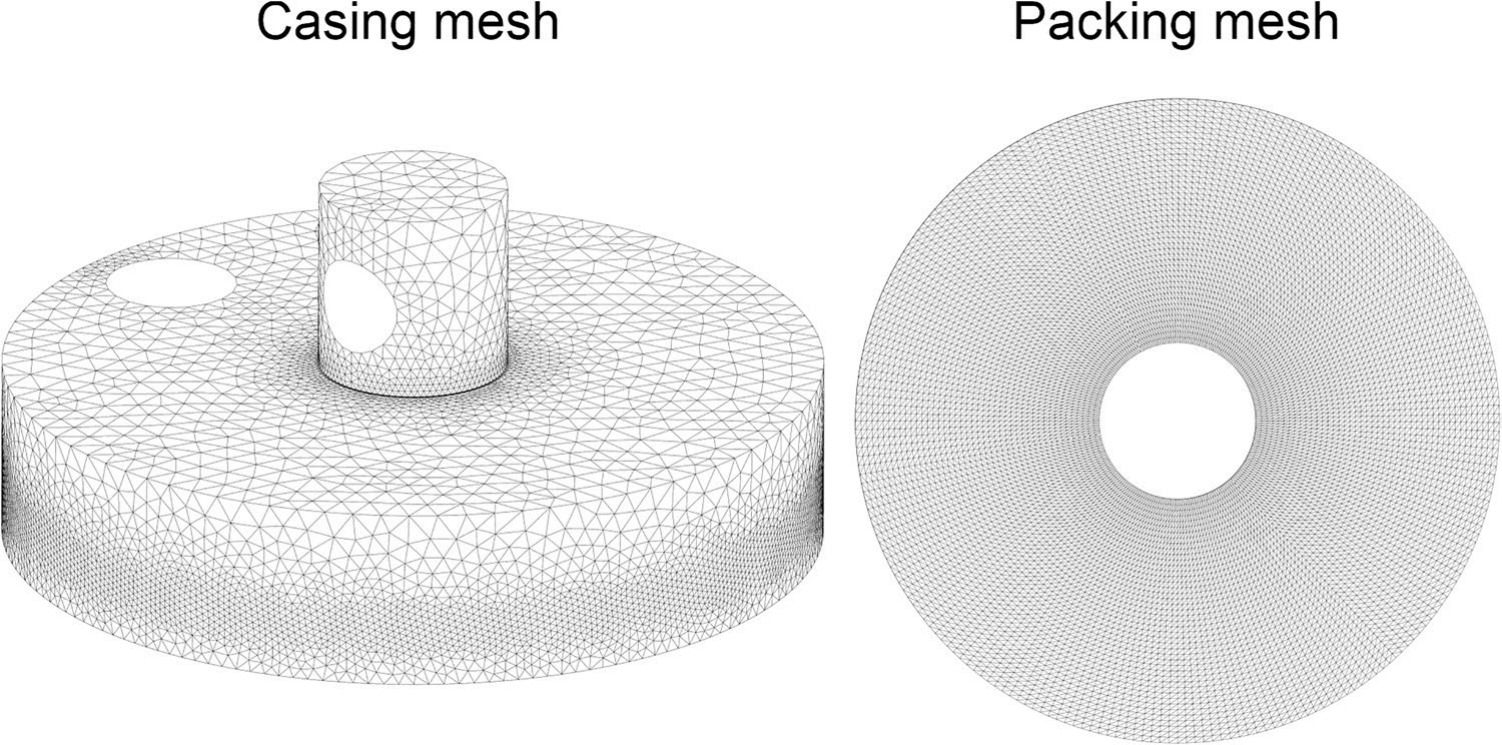
The final computational mesh used in RPB CFD simularions. Left- casing thetraedric mesh, Right - surface axisymmetric mesh of the rotor

Computational grid generation is a critical step that influences the convergence, stability and accuracy of the simulations. Therefore, a test of the independence of the result from the mesh density was performed. Figure 4 shows the results of CFD calculations of the gas pressure drop in the RPB device for different densities and mesh structures. At the beginning, a mesh was generated for a porous filling with cubic elements (cube). However, the lack of symmetry of the casing and quite a large jump in the size of the elements at the junction of the rotating and stationary domains resulted in the formation of elements with high skewness (the value of the average skewness of the elements is shown in parentheses in Fig. 4). Therefore, the shape of the element was changed to tetrahedrons, which allowed to improve the quality of the mesh cells. Five tetrahedral meshes with near wall boundary layers (wedge elements) of different element sizes were tested. Finally, a mesh with 1656K elements and 536K nodes with an average skewness of 0.33 was selected (Fig. 5). Further increasing the mesh density did not improve the result but only extended the computational time.

The obtained average skewness classifies the quality of the generated mesh as good. In order to improve the quality of a grid adjacent to the rotating components and the gas outlet, the mesh in these locations is significantly concentrated.

### Mathematical model

The classical modelling of turbulence is based on the Reynolds approach (RANS models—Reynolds-averaged Navier–Stokes models), for which the instantaneous values of all physical quantities characterising the flow at a given point in the flow area are the sum of the time-averaged quantities and the component of fluctuation (turbulence), which is a random function of time and space (Paszko and Łygas 2016). The use of this concept in the Navier-Stokes equation transforms it into the Reynolds equation, which for an incompressible fluid takes the form of Eq. 11:

11 $$\rho \left(\frac{\partial \overline{U_i}}{\partial t}+{\overline{U}}_j\frac{\partial \overline{U_i}}{\partial {x}_j}\right)=\frac{\partial }{\partial {x}_j}\left({\sigma}_{ij}\right)+\overline{F_i}$$

where *U* is the vector of velocity, *F* the force, *t* the time and *x* the linear dimension. The stress tensor (*σ*) then takes the form of Eq. 12:

12 $${\sigma}_{ij}=-\overline{p}{\delta}_{ij}+\overline{v}\rho \left(\frac{\partial \overline{U_i}}{\partial {x}_j}+\frac{\partial \overline{U_j}}{\partial {x}_i}\right)-\overline{\rho {v}_i{v}_j}$$

where *p* is the pressure. There is an additional term in the equation called the “Reynolds stress tensor“ *σ*_*T*_ (Eq. 13), a symmetric tensor, which in the case of a spatial system means that the values of six out of nine components are unknown (Paszko and Łygas 2016). This makes it impossible to close the system of equations and solve it using the analytical method.

13 $${\left({\sigma}_T\right)}_{ij}=-\overline{\rho {v}_i{v}_j}$$

In the literature, there are many models that try to solve the Navier–Stokes equations for turbulent flow using the iterative method. The most common turbulence model used in CFDs is the *k-ε* model, which belongs to the RANS group. It enables the solution of the Navier–Stokes equations by introducing two variables:

- Kinetic energy of turbulence (*k*)
- Kinetic energy dissipation rates (*ε*)

The *k-ε* turbulence model can be described by Eqs. 14 and 15 (Launder and Spalding 1974):

14 $$\rho \frac{dk}{dt}=\frac{\partial }{\partial x}\left[\left(\mu +\frac{\mu_t}{\sigma_k}\right)\frac{\partial k}{\partial x}\right]+{P}_k+{P}_b-\rho \varepsilon -{Y}_M+{S}_k$$

15 $$\rho \frac{d\varepsilon}{d t}=\frac{\partial }{\partial x}\left[\left(\mu +\frac{\mu_t}{\sigma_{\varepsilon }}\right)\frac{\partial \varepsilon }{\partial x}\right]+{C}_{1\epsilon}\frac{\varepsilon }{k}\left({P}_k+{C}_{3\varepsilon }{P}_b\right)-{C}_{2\varepsilon}\rho \frac{\varepsilon^2}{k}+{S}_{\varepsilon }$$

where *S* is the source. The turbulent fluid viscosity (*μ*_*t*_) is calculated from Eq. 16:

16 $${\mu}_t=\rho {C}_{\mu}\frac{k^2}{\varepsilon }$$

*P*_*k*_ is the source of turbulent kinetic energy related to velocity changes (Eq. 17):

17 $${P}_k=-\overline{\rho {v}_i{v}_j}\frac{\partial {v}_j}{\partial {x}_i}$$

*P*_*b*_ is the source of turbulent kinetic energy related to the elasticity of the fluid (Eq. 18):

18 $${P}_b=\beta {g}_i\frac{\mu_t}{{\mathit{\Pr}}_t}\frac{\partial T}{\partial {x}_i}$$

where *Pr*_*t*_ is the turbulent Prandtl number; in the standard model, *k-ε Pr*_*t*_ is constant (*Pr*_*t*_ = 0.85); *g*_*i*_ is the force of gravity in direction i; and *β* is the thermal expansion coefficient calculated from Eq. 19:

19 $$\beta =-\frac{1}{\rho }{\left(\frac{\partial \rho }{\partial T}\right)}_p$$

The constants in the *k-ε* model are:

- C_1ε_ 1.44
- C_3ε_ -0.33
- C_μ_ 0.09
- σ_k_ 1.0
- σ_ε_ 1.3

CFD solvers contain many implemented RANS models, including modifications to the *k-ε* model (RNG *k-ε* or Realizable k-ε), *k-ω*, SST *k-ω* and the most advanced Reynolds Stress Model. The turbulence model is selected individually for each problem.

The modelling of the rotating domain can be realised by the single reference frame (SRF) or multiple reference frame (MRF) approach. The SRF approach can refer to the entire computational domain (single moving reference frame). For more complex geometries, where stationary and rotating domains are simultaneously utilised, the MRF approach is applied. In this approach, it is very important to have well-defined interfaces between the stationary and rotating zones.

When the motion equations are solved in the rotating reference frame, the acceleration of the fluid is augmented by additional terms that appear in the momentum equations. Moreover, the equations can be formulated by expressing the momentum equations using (Batchelor 1967):

- Relative velocities as dependent variables (known as the relative velocity formulation)
- Absolute velocities as dependent variables in the momentum equations (known as the absolute velocity formulation)

For the relative velocity formulation, the governing equations of fluid flow for a steadily rotating frame take the form of consecutive equations: conservation of mass (Eq. 20), conservation of momentum (Eq. 21), and conservation of energy (Eq. 22) (Ansys 2009a).

20 $$\frac{\partial \rho }{\partial t}+\nabla \bullet \rho \overrightarrow{v_r}=0$$

21 $$\frac\partial{\partial t}\left(\rho\overrightarrow{v_r}\right)+\nabla\bullet\left(\rho\overrightarrow{v_r}\overrightarrow{v_r}\right)+\rho\left(\overrightarrow\omega\times\overrightarrow{v_r}+\overrightarrow\omega\times\overrightarrow\omega\times\overrightarrow r\right)=-\nabla p+\nabla\bullet\overset={\tau_r}+\overrightarrow F$$

22 $$\frac\partial{\partial t}\left(\rho E_r\right)+\nabla\bullet\left(\rho\overrightarrow{v_r}H_r\right)=\nabla\bullet\left(k\nabla T+{\overset=\tau}_r\bullet\overrightarrow{v_r}\right)+S_h$$

where *ω* is the angular velocity, *H*_*r*_ the relative total enthalpy, and *v*_*r*_ *the* relative velocity, *T* – temperature. In the momentum equation, we find two additional acceleration terms:

- The Coriolis acceleration ($2\overrightarrow{\omega\;}\;\times\;\overrightarrow{V_r}$)
- The centripetal acceleration ( $\overrightarrow{\;\omega}\times\overrightarrow\omega\times\overrightarrow r)$ )

The terms of the relative internal energy (*E*_*r*_) (Eq. 23) and the relative total enthalpy (Eq. 24), also known as rothalpy, are defined as:

23 $${E}_r=h-\frac{p}{\rho }+\frac{1}{2}\left({v}_r^2-{u}_r^2\right)$$

24 $${H}_r={E}_r+\frac{p}{\rho }$$

where *h* is the specific static (thermodynamic) enthalpy. For the absolute velocity formulation, the governing equations of fluid flow for a steadily rotating frame take the form of consecutive equations: conservation of mass (Eq. 25), conservation of momentum (Eq. 26) and conservation of energy (Eq. 27) (Ansys 2009a).

25 $$\frac{\partial \rho }{\partial t}+\nabla \bullet \rho \overrightarrow{v_r}=0$$

26 $$\frac\partial{\partial t}\left(\rho\overrightarrow{v_r}\right)+\nabla\bullet\left(\rho\overrightarrow{v_r}\overrightarrow v\right)+\rho\left(\overrightarrow\omega\times\overrightarrow v\right)=-\nabla p+\nabla\bullet\overset=\tau+\overrightarrow F$$

27 $$\frac\partial{\partial t}\rho E+\nabla\bullet\left(\rho\overrightarrow{v_r}H+p\overrightarrow{u_r}\right)=\nabla\bullet\left(k\nabla T+\overset=\tau\bullet\overrightarrow v\right)+S_h$$

In this formulation, the Coriolis and centripetal accelerations can be collapsed into a single term $\left(\overrightarrow{\omega}\times \overrightarrow{v}\right)$.

The problem of modelling porous media can be used to solve many different single-phase and multiphase problems, including flow through filled beds, filter papers and perforated plates.

The full porous model is both a generalisation of the Navier–Stokes equations and Darcyʼs law used to describe the flow in porous structures. Porous media are modelled by adding the momentum source component to the standard fluid flow equations. Calculation of the momentum for a porous element also depends on the type of material (isotropic or anisotropic). The model packings in this paper are isotropic; therefore, the equations describing the isotropic loss model in the x, y and z directions appear as follows (Ansys 2009a):

28 $${S}_{M,x}==-\frac{\mu }{K_{perm}}{U}_x-{K}_{loss}\frac{\rho }{2}\left|U\right|{U}_x$$

29 $${S}_{M,y}==-\frac{\mu }{K_{perm}}{U}_y-{K}_{loss}\frac{\rho }{2}\left|U\right|{U}_y$$

30 $${S}_{M,z}==-\frac{\mu }{K_{perm}}{U}_z-{K}_{loss}\frac{\rho }{2}\left|U\right|{U}_z$$

where *S*_*M*_ is the momentum source, *K*_*perm*_ the permeability and *K*_*loss*_ the loss coefficient. The first part of the equation (viscous component) is responsible for losses related to viscosity; the second part of the equation (inertial component) is responsible for losses related to inertia.

The described change in momentum contributes to the pressure gradient in the porous medium, which can be described by Eq. 31 (Ansys 2009b).

31 $$\Delta p=-{C}_1\Delta lv-{C}_2\Delta l{v}^2$$

The constants *C*_*1*_ and *C*_*2*_ appearing in the equation can be replaced by the equations containing *K*_*perm*_ and *K*_*loss*_ giving Eq. 32 (Ansys 2009b).

32 $$\Delta p=-\frac{\mu }{K_{perm}}\Delta lv-\frac{\rho {K}_{loss}}{2}\Delta l{v}^2$$

Therefore, the coefficients *K*_*perm*_ (Eq. 33) and *K*_*loss*_ (Eq. 34) look like this (Ansys 2009b):

33 $${K}_{perm}=\frac{\mu \Delta l}{C_1}$$

34 $${K}_{loss}=\frac{2{C}_2\Delta l}{\rho }$$

The determination of the above coefficients from the obtained relationship between pressure drop and fluid flow can be performed experimentally or by using additional numerical calculations. The obtained coefficients allow for a good description of the porous medium in the Ansys program and, thus, result in reliable data by simulation.

### Boundary conditions

**Fig. 6:**
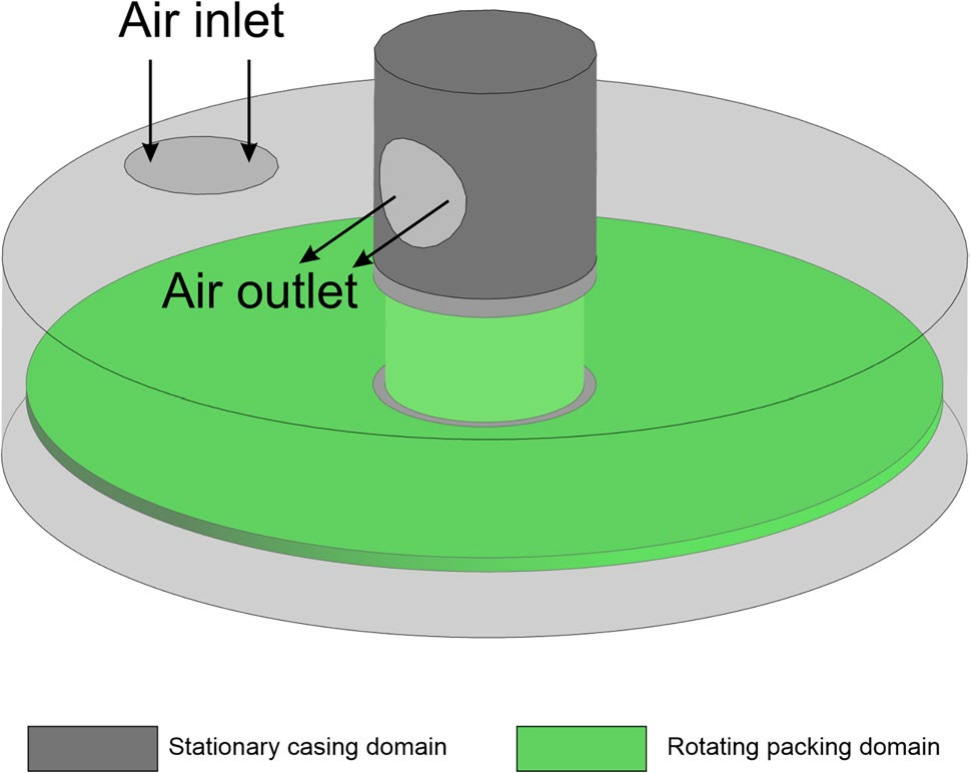
View of the geometry of the CFD model with the gas inlet and outlet marked

An important factor that characterises the operation of the RPB device is the rotating filling inside the housing. Therefore, it was necessary to simulate the flow on a dynamic mesh by defining the rotational elements of the model and giving them a specific rotational frequency. The *velocity inlet* boundary condition was used as the air inlet. The velocity inlet boundary condition was used as the air inlet. The application of this boundary condition was dictated by the method of measuring the air flow, which returned the value in m^3^/h, which was a value independent of the operating temperature of the apparatus. The domain exit condition was set to a *pressure outlet* with an operating pressure of 1 atmosphere. The geometry view with the gas inlet and outlet marked is shown in Fig. 6.

In this work, calculations of pressure drop for various conditions of single-phase (gas) flow through the RPB device with rotating packing were performed. For comparison, the parameters of the dry pressure drop test in the simulation were the same as the experimental conditions. Based on the obtained data, the numerical model was validated. The simulation performed calculations for two turbulence models to determine which one better and reflected the experimental results compared to the required calculation time.

Basic model parameters:

- Porosity: 0.922
- Interface size: 2800 m^2^ m^-3^
- Type of porous filling: isotropic metal foam
- Turbulence model: k-ε, RNG k-ε
- Rotational frequencies: 150–1500 rpm
- Volumetric gas flow rate: 20 m^3^ h^-1^, 40 m^3^ h^-1^, 60 m^3^ h^-1^
- No liquid flow
- The system is isothermal and the gas is incompressible

The obtained results for the presented basic model parameters were unsatisfactory; therefore, it was necessary to validate the model by introducing parameters describing the fill properties that determine momentum loss. The loss of momentum by an isotropic porous region can be described by parameters such as permeability (*K*_*perm*_) and loss factor (*K*_*loss*_).

**Fig. 7:**
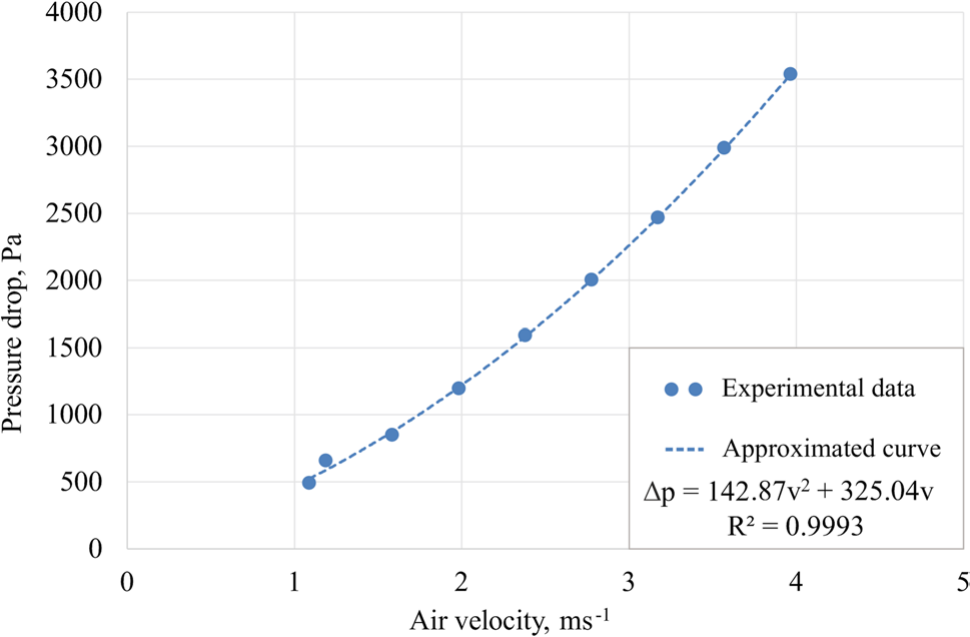
Gas pressure drop through stationary filling depending on the gas velocity

**Fig. 8:**
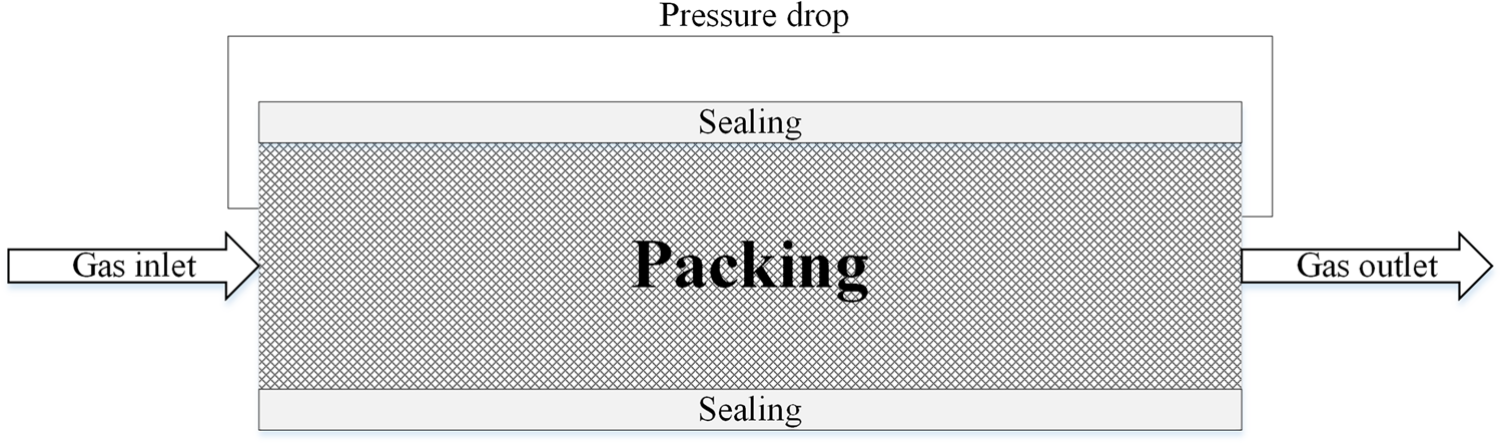
Experimental configuration for measuring the filling parameters for the CFD model

In order to obtain the values of permeability and loss coefficient, additional experiments were carried out showing the dependence of the pressure losses through the filling fragment on the gas flow velocity. The obtained dependence is presented in Fig. 7. A stationary RPB device was used to perform the measurements, and two tightly insulated pieces of filling were placed inside the rotor (Fig. 8). The *K*_*perrm*_ and *K*_*loss*_ parameters calculated based on the relationships presented above were:

- Permeability (*K*_*perm*_): 2.58 ∙ 10^-8^ m^2^;
- Loss factor (*K*_*loss*_): 2744.11 m^-1^.

### Experimental design

In this study, a single-stage RPB unit was investigated (Fig. 2). To conduct experiments, the metal nickel-chromium foam packing (NCX2733, Recemat BV, Netherlands) with an outer diameter of 600 mm, a height of 10 mm and an inner packing diameter of 146 mm was used. The rotor plates are connected to each other by bolts, between which there are additional spacer rings, which ensure a constant height of the gap between the rotor plates. The internal diameter of the RPB casing is 632 mm. So, for a rotor with a diameter of 600 mm, the distance between the inner radius of the casing and the radius of the rotor is 16 mm. Experiments in this study were conducted according to the following operating conditions:

- Packing porosity: 0.922
- Packing interface size: 2800 m^2^ m^-3^
- Rotational frequencies: 150 rpm, 300 rpm, 450 rpm, 600 rpm, 900 rpm, 1200 rpm, 1500 rpm
- Volumetric gas flow rate: 20 m^3^ h^-1^, 40 m^3^ h^-1^, 60 m^3^ h^-1^
- No liquid flow

Each measurement point was performed with the setpoint gas flow rate and rotor speed set. After passing the given point to the experimental point, it was waited for the parameters to stabilize and then the result was recorded. Each measurement was repeated three times.

## Results and discussion

In this study, single-phase (gas phase) flow in RPB with porous packing was investigated. Experiments were performed for seven rotational frequencies from 150 rpm to 1500 rpm. The measurement error was ± 15 Pa. The relative error was calculated for both experimental and simulation results (Eq. 35).

35 $$\mathrm{relative}\ \mathrm{error}\ \left[\%\right]=\frac{\left| experimental\ value- simulation\ value\right|}{experimental\ value}\bullet 100\%$$

**Fig. 9:**
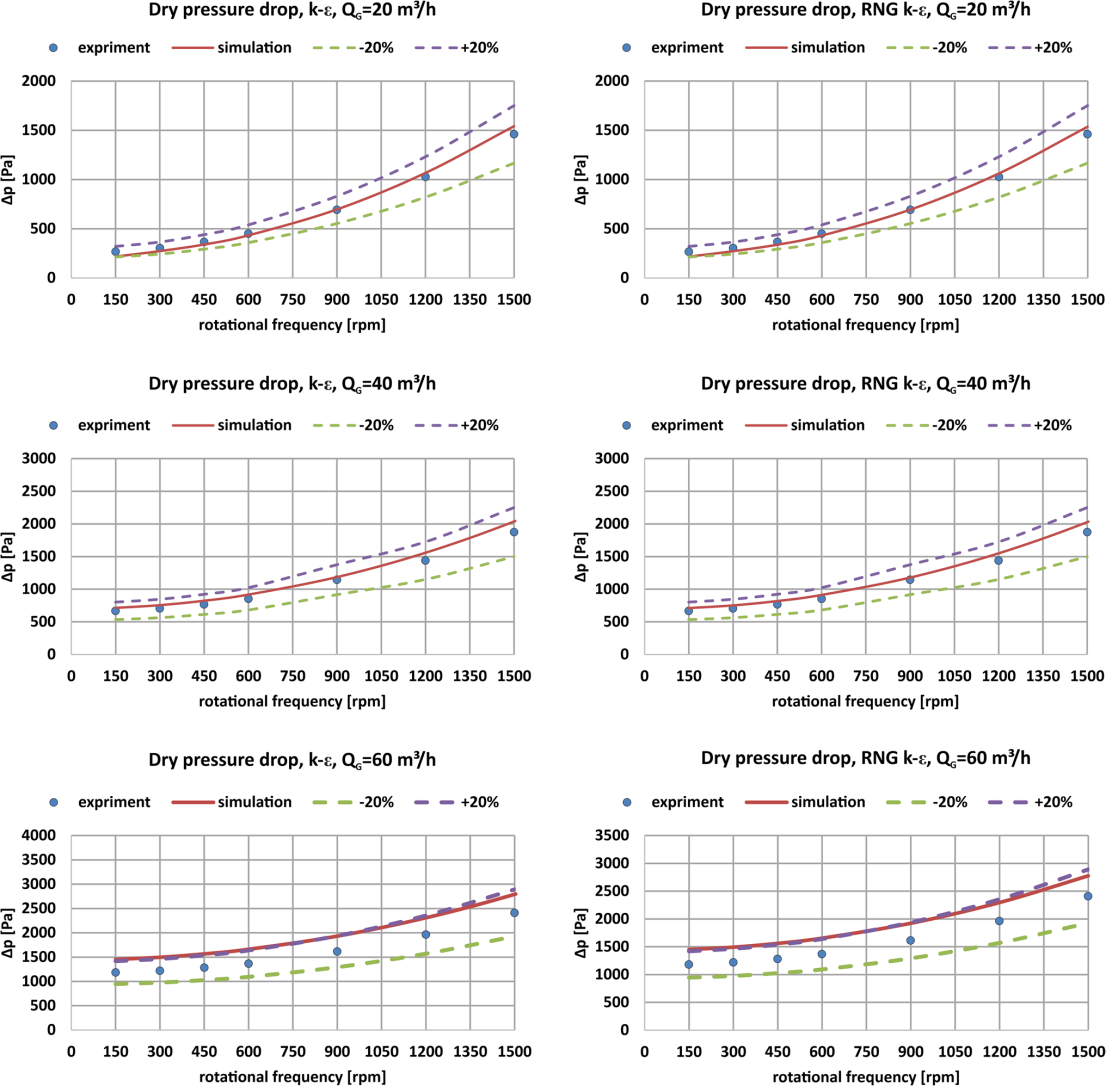
Dry pressure drop in the RPB device for experimental data and CFD simulation results for the k-ε the RNG k-ε models in the rotational frequency range 150–1500 rpm and gas flow rates 20 m^3^ h^-1^, 40 m^3^ h^-1^ and 60 m^3^ h^-1^

The comparison of the simulation and experimental results for the fluid models k-ε and RNG k-ε, as well as for the respective gas flow rates, 20 m^3^ h^-1^, 40 m^3^ h^-1^, 60 m^3^ h^-1^, is presented in the figures below Fig. 9.

The obtained results indicate a very good consistency of the results obtained by CFD modelling with the experimental results for all analysed gas flow rates giving a relative error less or equal than ± 20%. The greatest discrepancy between the simulation and experimental results is noted for gas flow rate 60 m^3^ h^-1^; however, it still gives satisfactory results considering the relative error. With increasing gas flow rate the relative error increases, but the error value is still lower than 20%. What is more, the shape of the simulation curve has the same character as the experimental results. This indicates a good preparation of the model and allows for the continuation of work related to the modelling of processes using numerical methods.

The k-ε and RNG k-ε turbulence models do not show significant differences in the obtained results for pressure drop (<10 Pa); however, it should be noted that the calculations using the RNG k-ε model are more time-consuming due to its more complex character, which includes the effect of swirl on turbulence, enhancing accuracy for swirling flows. A test simulation was also performed using the Reynolds Stress model, which showed no difference in the result with respect to the standard k-ε. However, the computation time was unacceptably long; therefore this model was not used in further analyzes. Therefore, the classic k-ε model is recommended for further work because it gives satisfactory simulation results in less time.

The experimental pressure drop results indicate that both the increase of the gas flow rate and the rotational frequency causes increase in the dry pressure drop value.

Table 1 presents a summary of all the results obtained in the experiments, the CFD simulations for the k-ε and RNG k-ε models and the relative errors obtained for the simulation in each calculation variant.

**Table 1:** Experimental and calculated dry pressure drops for NC2733 metal foam with a diameter of *d* = 600 mm

m^3^ h^-1^	Rotational frequency	Δp experimental	Δp k-ε model	Relative error	Δp RNG k-ε model	Relative error
	rpm	Pa	Pa	%	Pa	%
20	150	266.50	217	18.57%	216.81	18.65%
	300	303.50	272.83	10.11%	271.93	10.40%
	450	366.00	339.73	7.18%	338.25	7.58%
	600	449.00	432.76	3.62%	430.86	4.04%
	900	692.00	696.37	0.63%	694.83	0.41%
	1200	1026.50	1067.24	3.97%	1062.64	3.52%
	1500	1459.50	1541.72	5.63%	1536.79	5.30%
40	150	668.00	712.75	6.70%	712.39	6.65%
	300	704.50	754.56	7.11%	753.29	6.93%
	450	768.00	822.44	7.09%	820.22	6.80%
	600	852.50	916.71	7.53%	913.59	7.17%
	900	1145.50	1185.02	3.45%	1179.69	2.98%
	1200	1437.50	1558.44	8.41%	1551.40	7.92%
	1500	1875.00	2038.10	8.70%	2029.31	8.23%
60	150	1182.00	1452.06	22.85%	1452.40	22.88%
	300	1220.00	1496.06	22.63%	1494.71	22.52%
	450	1282.00	1565.45	22.11%	1562.66	21.89%
	600	1366.50	1660.85	21.54%	1656.60	21.23%
	900	1615.00	1930.59	19.54%	1924.05	19.14%
	1200	1962.00	2307.61	17.62%	2296.87	17.07%
	1500	2409.50	2789.82	15.78%	2776.71	15.24%

**Fig. 10:**
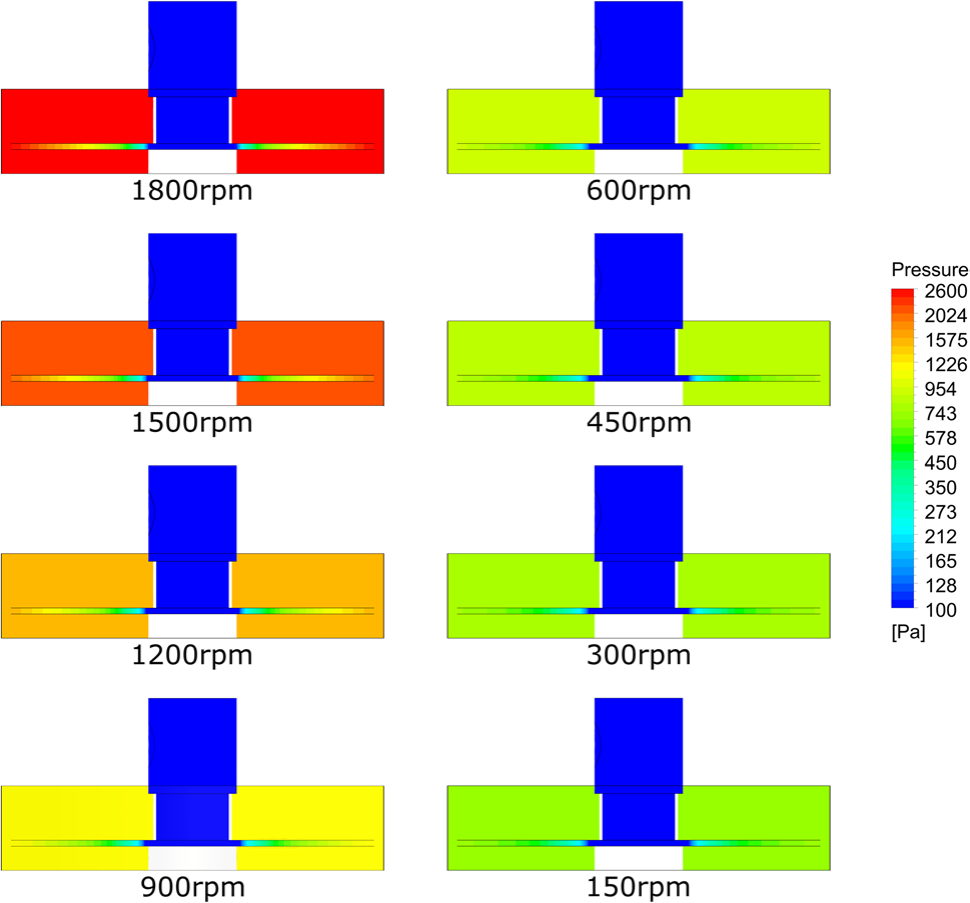
CFD simulation results static overpressure distribution in cross section of the RPB device for different packing rotation speed. Simulations preformed for the = 40m^3^/h

The analysis of the CFD simulation results shows that the increase in pressure in the bed housing for a constant gas flow depends mainly on the rotor speed (Fig. 10). The rotary motion produces inertial forces (in this case centrifugal force) acting along the radius in the direction opposite to the gas flow. Increasing the rotation of the filling causes an increase in pressure in the RPB housing, which causes the need to increase the efficiency of the supply fan. It is worth noting that for the tested filling, the influence of rotation on the pressure increase in the range of 150–600 rpm is small. Only increasing the rotor speed above 600 rpm causes a more than threefold increase in pressure drops.

**Fig. 11:**
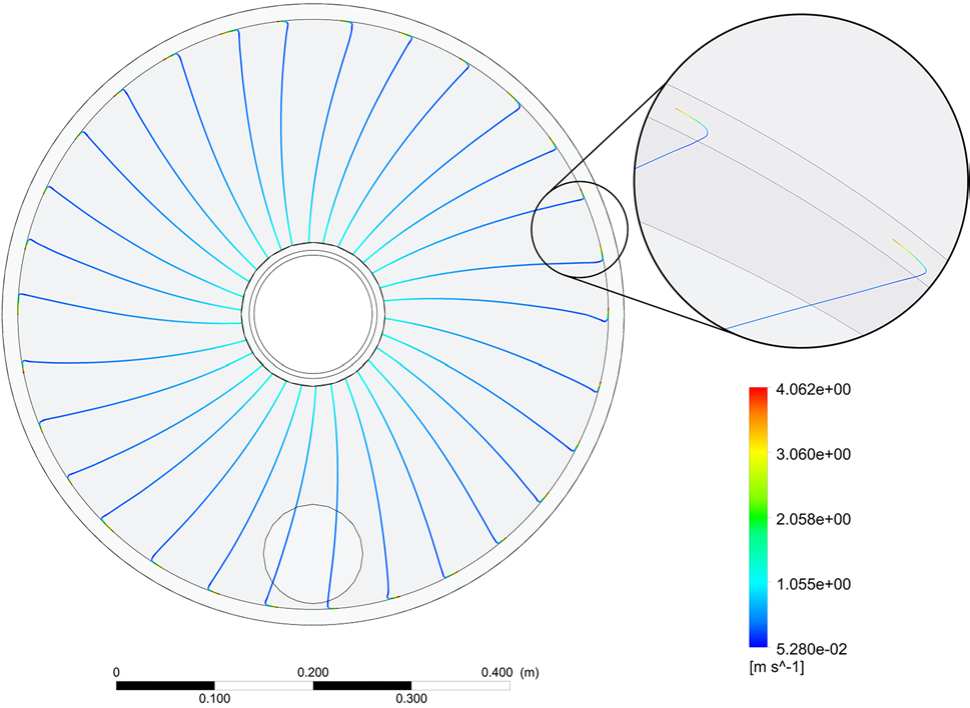
Gas flow lines in the RPB device for the rotor speed of 900 rpm and the volumetric gas flow rate of 60 m^3^ h^-1^

Figure 11 shows the lines of gas flow through a packing rotating at 900 rpm. Based on the obtained results, it can be observed that at the gas inlet to the rotor, the gas entrainment effect is noticeable, which causes a slight bending of the flow trajectory due to gas inertia. This, however, does not extend the gas residence time inside the rotating packing. Further, the gas moves radially through the packing to the outlet. The rest of the investigated variants show the same pattern of gas flow streamlines through the packing. What is more, with increasing rotational speed of the packing, air velocity at the inlet to the packing increases and the entrainment effect is stronger.

The effect of the influence of rotation on the hydrodynamics of gas flow through the rotating bed and the related pressure drops can be described by means of CFD calculations. The pressure drops caused by the bed will increase with the addition of the liquid phase. It is therefore essential to check for dry pressure drops in order to select the fan in advance or determine the optimal gas flow parameters.

**Fig. 12:**
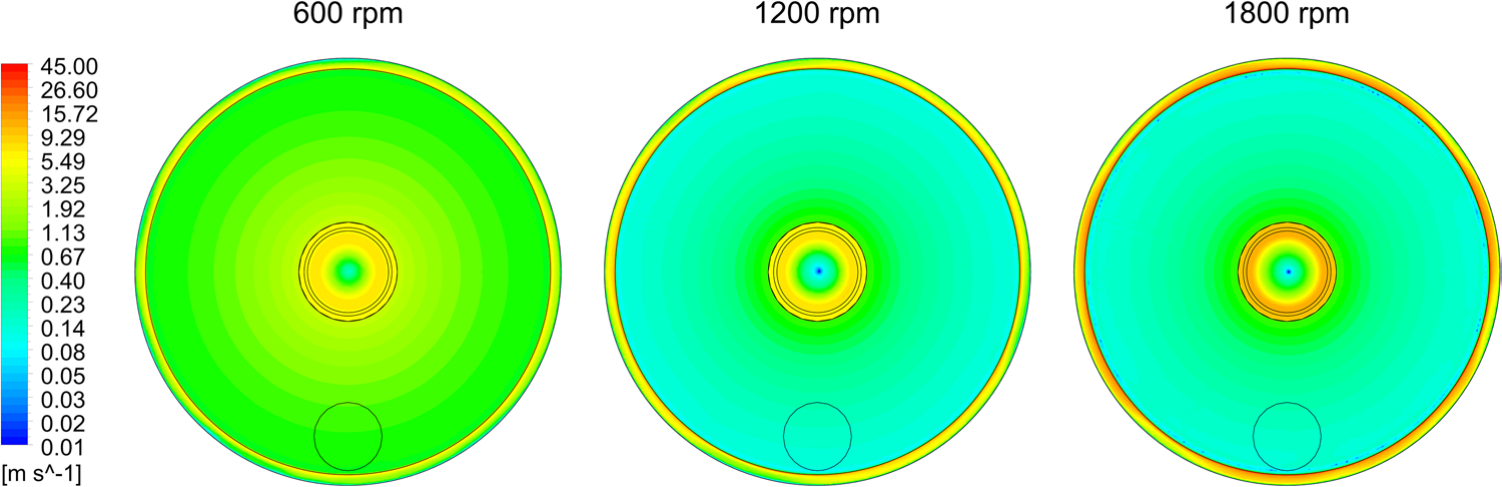
Comparison of gas velocity distributions inside the RPB device for three selected rotor speeds of 600, 1200 and 1800 rpm. The volumetric gas flow rate of 40 m^3^ h^-1^

The high pressure drop inside the rotating packing causes the gas velocity gradients to equalize along the bed radius (Figure 12). This effect minimizes the asymmetry of the device caused by the side location of the gas inlet. Although the gas is introduced parallel to the axis of rotation, it quickly gains angular velocity and rotates in the absorber housing. As a result of inertia, the air swirls leaving the absorber, which is visible by the formation of a low velocity zone in the axis of the gas outlet (absorber center). Such strong swirling of the gas in the center of the absorber can prevent effective spraying of liquid onto the bed during operation of the RPB apparatus. Therefore, it is necessary to find a range of operational parameters in which, as a result of rotation, the liquid will be well distributed over the bed and the interfacial exchange surface developed to the maximum while minimizing the gas pressure drops in the device.

## Conclusions

A 3D model was built to investigate the gas phase flow behaviour in a rotating packed bed. The parameters of pressure drop through porous domains were selected experimentally. These parameters were then used in CFD simulations. The model has been verified by comparing the results obtained in simulations with experimental results for the same parameters.

In this study, two fluid models were investigated (k-ε and RNG k-ε). The results for both models give very good consistency of the simulation results with the experimental ones. The RNG k-ε model had significantly longer computational time and gave no significant advantages over the simulation results compared to the k-ε model. CFD is a powerful tool to predict gas phase behaviour in RPB with porous packing. What is more, the CFD can be used to predict the hydrodynamics of new types of packing and provides validation in order to lower pressure drop and improve fluid pattern inside the packing.

The error between the experimental and simulation results increases with increasing gas flow rate. This may be due to errors in the fit of the curve used to determine the permeability and loss coefficient, in which velocity is squared. Therefore, the error in matching the experimental with the simulation results increases significantly with increasing gas flow rate.

The entrainment effect only occurs in a small section at the inlet of the gas phase to the packing. It results from the entry of the gas phase from the casing to the packing. It has been noticed that the entrainment effect is stronger as rotor speed increases. This behaviour is due to the increased inertia of the gas, which increases with the rotor speed. After a strong curvature at the entry of the gas streamline, it stabilises further.

The results show that the CFD model is effective in modelling gas phase behaviour in RPB units equipped with porous packing. The understanding of one phase and two phase flow behaviour inside RPB units it is still insufficiently understood and described. It is necessary to deepen the research and CFD simulations related to the analysis of the behaviour of the phases in the RPB device. This model is a good basis for further work on CFD simulations of two-phase flows in the RPB, which are a very complex issue.

## Data Availability

All measurements and data analyses have been made in accordance with applicable standards and the software used for simulation and data processing comes from a legal source and has a current user license allowing for scientific publications. The datasets generated during and/or analysed during the current study are available from the corresponding author on reasonable request.

## Nomenclature

C: constant
c_f_: skin friction coefficient; -
E_r_: relative internal energy
F: force; N
G: growth step; -
g: gravity force; m s^-2^
h: specific static (thermodynamic) enthalpy; m^2^ s^-2^
H: absolute total enthalpy; m^2^ s^-2^
H_r_: relative total enthalpy; m^2^ s^-2^
k: turbulent kinetic energy; m^2^ s^-2^
K_loss_: loss coefficient; m^-1^
K_perm_: permeability; m^2^
l: length or characteristic dimension; m
N: number of layers; -
p: pressure; Pa
P: turbulent kinetic energy; kg m^-1^ s^-3^
Pr: Prandtl number; -
r: location vector; m
Re: Reynolds number; -
S: additional source terms
S_M_: momentum source; kg m^-2^ s^-2^
t: time; s
T: temperature; K
U: vector of velocity; m s^-1^
y_H_: height of the 1^st^ cell element; m
v: velocity; m s^-1^
v_r_: relative velocity; m s^-1^
${\dot{\mathrm{V}}}_{\mathrm{G}}$: gas flow rate; m^3^ h^-1^
x: linear dimension; m

## Greek letters

*β*: thermal expansion coefficient; K^-1^
*ρ*: density; kg m^-3^
*δ*: thickness of the laminar boundary layer; m
*σ*: stress tensor
*μ*: dynamic viscosity; kg s^-1^ m^-1^
*ε*: kinetic energy dissipation rate; m^2^ s^-3^
*τ*: shear stress; kg m^-1^ s^-2^
*ϑ*: kinematic viscosity; m^2^ s^-1^
*ω*: angular velocity; s^-1^

## Abbreviation

CFD: Computational Fluid Dynamics
HiGee: High gravity
MRF: multiple reference frame
RANS: Reynolds-averaged Navier–Stokes
RPB: Rotating packed bed
SRF: single reference frame
